# Comparison of four value sets derived using different TTO and DCE approaches: application to the new region-specific PBM, AP-7D

**DOI:** 10.1186/s12955-024-02233-2

**Published:** 2024-02-10

**Authors:** Takeru Shiroiwa, Tatsunori Murata, Yasuhiro Morii, Eri Hoshino, Takashi Fukuda

**Affiliations:** 1 Center for Outcomes Research and Economic Evaluation for Health (C2H), National Institute of Public Health, 2-3-6 Minami, Wako, Saitama 351-0197 Japan; 2 Crecon Medical Assessment Inc, 2-12-15, Shibuya, Tokyo, Shibuya-Ku 150-0002 Japan; 3 Division of Policy Evaluation, Department of Health Policy, Research Institute, National Center for Child Health and Development, 2-10-1 Okura, Setagaya-Ku, Tokyo, 157-0074 Japan

**Keywords:** Preference-based measure, QALY, AP-7D, TTO, DCE

## Abstract

**Background:**

AP-7D is a newly developed preference-based measure (PBM) in East and Southeast Asia. However, no value set has been established yet. Comparison of the characteristics of value sets obtained by different methods is necessary to consider the most appropriate methodology for valuation survey of AP-7D.

**Method:**

We surveyed the general population’s preference of AP-7D health states by four valuation methods (a) composite time trade-off (cTTO); (b) simple discrete choice experiment (DCE); (c) DCE with duration; and (d) ternary DCE. In Japan, we collected approximately 1,000 samples for cTTO tasks through a face-to-face survey and 2,500 samples for each of the three DCE tasks. Respondents were selected through quota sampling based on the sex and age. The cTTO data were analyzed using a linear mixed and tobit model; the DCE data were analyzed using a simple and panel conditional logit model. Where the results of the analysis showed inconsistencies, a constrained model was used.

**Results:**

Since all the unconstrained models, except simple DCE, showed one or more inconsistencies, the constrained model was used for the analyses. The minimum values for the models were as follows: TTO model, -0.101; simple DCE model, -0.106; DCE with duration model, -0.706; ternary DCE model, -0.306. The score for the DCE with the duration model was much lower than that for the other models. Although the value sets for AP-7D differed among the four valuation methods, the ternary DCE model showed intermediate characteristics between those of the cTTO and DCE with duration models. As compared with to EQ-5D-5L, the distributions of all the scores on the Japanese AP-7D moved to the left. Although “Energy” was one of the domains with the least influence on the AP-7D score in all four models, “Burden to others” had the largest impact on the preferences.

**Conclusion:**

We constructed four value sets using different TTO and DCE methods. Our findings are expected not only to contribute to the development of AP-7D, but also other preference-based measures.

**Supplementary Information:**

The online version contains supplementary material available at 10.1186/s12955-024-02233-2.

## Introduction

Economic evaluations to measure the outcomes of healthcare technologies are often based on calculation of the quality-adjusted life-years (QALYs). Public health technology assessment (HTA) agencies normally recommend its use for cost-effectiveness analysis. For example, in Japan, new HTA systems for drug and medical-device pricing were introduced in 2019 [1]. Preference-based measures (PBMs) are generally used to measure the utilies of health states, which can then be used for calculating QALYs. PBMs, such as a general PBM [2–6], PBM for pediatric and/or adolescent people [7, 8], disease-specific PBM [9, 10], and PBM for social care [11, 12] have also been developed. However, until now, these PBMs have mainly been developed for Western countries; for example, the EQ-5D was developed in Europe, HUI in Canada, 15D in Northern Europe, AQoL in Australia, and SF-6D, ASCOT, and CHU-9D in the UK.

Considering this situation, we developed a new Asia-preference-based measure-7 dimension (AP-7D) [13] (Additional file 1) based on an interview survey and qualitative analysis of data from nine Asian countries (i.e., Indonesia, Japan, Korea, mainland China, Malaysia, the Philippines, Singapore, Taiwan, and Thailand). The AP-7D was developed to reflect the important concepts of East and Southeast Asians for utility measurements, collaborating between HTAsiaLink and ​Center for Outcomes Research and Economic Evaluation for Health (C2H) in Japan.

After the new PBM was developed, we needed to construct a value set of AP-7D for every country. The value set might differ between countries because of differences in culture, population characteristics, and potential issues with questionnaire translation. Therefore, it is important to develop value sets in each country and compare them across countries to better understand the differences in preferences for the AP-7D states among countries or regions. However, currently, we lack a methodology for appropriately evaluating the health states of AP-7D. Some standard methods are used to construct value sets in valuation surveys. Time trade off (TTO), discrete choice experiments (DCE), DCE with duration, and ternary DCE are typical examples of valuation methods. There is no consensus on which the most appropriate method might be, as it depends on the characteristics of the PBM. Thus, in this study, we constructed the first preliminary value sets for AP-7D in Japan, and compared four valuation methods to consider the most appropriate methodology for valuation survey of AP-7D.

## Methods

### AP-7D

The AP-7D was co-developed by ​HTAisaLink and the Center for Outcomes Research and Economic Evaluation for Health (C2H), National Institute of Public Health (NIPH) in Japan, and was established based on East and Southeast Asian concepts of health and health-related impacts. Our new PBM comprises seven domains: pain/discomfort (PD), mental health (MH), energy (EN), mobility (MO), work/school (WS), interpersonal interactions (II), and burden to others (BO), each of them classified on a four-grade scale (not at all, a little, quite a bit, and very much). AP-7D was originally developed in English and then translated into eight local languages. The instrument is shown in the Supplement.

### Composite TTO, Simple DCE, DCE with duration, and ternary DCE

We evaluated the AP-7D health states using the composite TTO (cTTO) [14], simple DCE [15], DCE with duration, and ternary DCE methods [16]. The TTO survey respondents always began with a conventional TTO task, i.e., living for 10 years in a health state described by the AP-7D, or living for x years in full health. If they considered the presented AP-7D state to be better than immediate death (i.e., x > 0), the value of x was varied until indifference was reached and the value of the AP-7D state was x/10. If the participants considered immediate death to be better than living for 10 years in the AP-7D state (i.e., x < 0), a lead time TTO [17] was started, which allowed estimation of negative values. In lead-time TTO, a set of choices is offered between “y years of life in full health” and “10 years in sound health followed by 10 years in the presented AP-7D state”. The value of y was varied until indifference was reached and the value of the AP-7D state was (y-10)/10.

The DCE method presented two health states (A and B) described by AP-7D. In the case of DCE with duration and ternary DCE, expected life-years (1, 4, 7, and 10 years) were combined with the AP-7D description. In the simple DCE and DCE with duration methods, the respondents chose the option they preferred between the two given choices. In the ternary method, three health states (state A, state B, and “immediate death”) were shown to the respondents, and they were asked to identify what they believed were the best and the worst health states.

### Face-to-face survey for cTTO

A face-to-face survey was conducted to collect the cTTO data. Respondents (aged 20–69 years) were recruited through a panel owned by a research company, based on non-random quota sampling by sex and age. Those aged 20–69 years were included. As it was challenging to recruit elderly people for this survey during the COVID-19 outbreak considering a high risk for contracting COVID-1, respondents aged > 69 years could not be recruited for valuation of AP-7D.

The target sample size was approximately 1,000. This was not based on the number of subjects included in the EQ-5D-5L valuation survey. The respondents were asked to visit a survey center in Tokyo. Computer-assisted personal interviews (CAPI) was performed with the interviewers’ support in a one-on-one, 60-min session at the survey center.

We prepared 14 blocks, and each block included 8 cTTO tasks based on an orthogonal design. The block by orthogonal design was generated by Ngene, which considers D-error minimization. Each respondent was randomly allocated to one block. The three training TTO tasks were completed before the actual TTO tasks [18]. The health states for the block were shown in random order. Responses were automatically collected as electronic data.

### Online survey for DCE

An online survey was conducted to collect DCE data, including simple DCE, DCE with duration, and ternary DCE. Respondents (aged 20–69 years for consistency with the face-to-face population) were recruited through a Japanese web panel, based on quota sampling by sex and age. The target sample number was approximately 2,500 for each of the DCE valuation methods, namely, simple DCE, DCE with duration, and ternary DCE. Each block had 15 pairs, and each respondent was randomly allocated to 10 blocks, based on the D-Optimal design methods in NGene. The health state pairs in the block and position of the cards (left or right) were shown in random order to prevent ordering and positioning effects.

### Statistical analysis

We calculated the numbers and percentages for the background factors, which were then compared with the norm data. The total time taken to complete all the 8 TTO or 15 DCE tasks was also calculated.

- a) cTTO
  Responses to the TTO task were converted into TTO scores as described in the subsection of”Composite TTO, simple DCE, DCE with duration, and ternary DCE”. The data were analyzed using a linear mixed model with “1-utility” as the dependent variable. The constant term and dummy variables representing the levels of the seven dimensions (7 × [4 − 1] = 21) were treated as fixed effects, and the respondents were treated as random effects. Interaction with any level 4 responses was considered by adding the N4 term (N4 = 1, if any level 4 responses were included in the health states) to the normal linear mixed model. The N34 term was also similarly defined (N34 = 1, if any level 3 or 4 responses were included in the health states) to consider the effects on the worst health states, which were observed in the EQ-5D-3L and -5L valuation surveys in a few countries. In addition, the TTO score was censored at 1. Considering these distribution characteristics, the Tobit model was also used for the cTTO data.
- b) Simple DCE
  The DCE data were analyzed using a simple and panel conditional logit model with the same 21 dummy variables as in the cTTO model. Similar to the case in the cTTO analysis, N4 and N34 terms were also considered in the conditional logit model. These analyses extracted the latent coefficients for AP-7D scoring. The DCE latent “dis-score,” defined as the sum of the latent DCE coefficients for each health state, was converted to the utility scale.
  To convert the latent DCE scores to a scale anchored at full health (1) and death (0), the modeled DCE values were anchored using the observed cTTO values. The linear relationship function between the mean latent DCE scores and mean cTTO values of the 112 health states measured in this face-to-face survey were estimated. Finally, the DCE coefficients were transformed by the estimated linear mapping function.
- c) DCE with duration and ternary DCE
  A simple and panel conditional logit model with or without N4 or N34 interactions was used to analyze the choice tasks, similar to the case for the simple DCE data. In the case of ternary DCE, a task was separated into two dichotomous choices and in the immediate death profile, the duration was treated as 0. For both types of the DCE data, the model for the estimation of coefficients was based on Bansback et al. [19] and included continuous duration (time) as well as interaction between the duration and each domain. Assuming t to be the duration and u_ij_ to be the utility of profile j for individual i, u_ij_ can be formulated as follows:
  $$\mathrm{U_{ij} }=\upbeta _{1}\mathrm{t_{ij} }+ {\varvec{\upbeta}}_{2}\mathbf{x}\mathrm{_{ij}t_{ij} }+\mathrm{\varepsilon _{ij}}$$

where ε_ij_ denotes the error term. However, the estimated **β**_2_, which indicates the vector of all the DCE coefficients in each domain, is not anchored to death (0) or full health (1). To change the latent coefficients to the disutility of each level, we divided the ratio of estimated **β**_2_ (vector) by the coefficients of time (β_1,_ scholar).

If the estimated disutility was not consistent (consistency implied that “weights at the higher level in the same domain were higher and those at the lower level were lower”), inconsistent levels were combined and was similarly analyzed by the same model (“constrained” model).

These analyses were performed using SAS 9.4 and Stata 17.

## Results

The collected sample included 1,050 respondents for the cTTO tasks; 2,725 respondents for the simple DCE tasks; 2,739 for the DCE with duration tasks; and 2,742 for the ternary DCE tasks. Thus, we were able to collect more samples than planned. The median total response time of the respondents to the eight TTO questions was 19.8 min (interquartile range (IQR) 17.5–23.0 min), to the 15 DCE questions was 7.1 min (IQR 4.5–10.8 min), to the 15 DCE with duration questions was 7.7 min (IQR 4.8–12.1 min), and to the 15 ternary DCE questions was 8.2 min (IQR 5.2–13.5 min). TTO tasks, based on face-to-face tasks, require more time than DCE web-based tasks. The response times for the DCE with duration and ternary DCE tasks were longer than those for the simple DCE tasks.

### Demographic factors

Table 1 shows the background characteristics of the respondents. The actual percentages of population by age category are 10.1% (aged 20–29), 10.9% (30–39), 13.9% (40–49), 14.0% (50–59), and 12.0% (60–69). We used the same weight of every age category for sampling, because equality of weight between generations should be reflected. The median household income ranged from JPY 5 to 7 million. As compared with the average household income of all Japanese families of JPY 5.6 million in 2021 [20], the household income was slightly higher. According to the 2019 Labour Force Survey, [21] full-time and part-time workers accounted for 31.6% and 13.7%, respectively. In total, 24.3% of Japanese individuals had graduated from university or graduate school in 2017, and 61.3% and 31.6% were married and unmarried, respectively, in 2015. Thus, the characteristics were comparable to the observations in the general population. However, as the respondents were recruited based on non-random sampling, the differences may influence the results.

**Table 1:** Background factors

	(a) TTO		(b) simple DCE		(c) DCE with duration		(d) Ternary DCE	
	Number	Percentage	Number	Percentage	Number	Percentage	Number	Percentage
	*N* = 1050		*N* = 2725		*N* = 2739		*N* = 2742	
Sex								
Male	525	50.0%	1361	49.9%	1368	50.0%	1356	49.5%
Female	525	50.0%	1364	50.1%	1371	50.1%	1386	50.6%
Age (years)								
20–29	210	20.0%	540	19.8%	536	19.6%	537	19.6%
30–39	210	20.0%	540	19.8%	550	20.1%	545	19.9%
40–49	210	20.0%	546	20.0%	547	20.0%	557	20.3%
50–59	210	20.0%	544	20.0%	546	19.9%	544	19.8%
60–69	210	20.0%	555	20.4%	560	20.5%	559	20.4%
Employment								
Full-time worker	682	65.0%	1243	45.6%	1215	44.4%	1249	45.6%
Part-time worker	142	13.5%	409	15.0%	402	14.7%	438	16.0%
Self employed	51	4.9%	197	7.2%	196	7.2%	183	6.7%
Housemaker	126	12.0%	430	15.8%	462	16.9%	466	17.0%
Retired	6	0.6%	239	8.8%	251	9.2%	239	8.7%
Student	42	4.0%	155	5.7%	153	5.6%	112	4.1%
Other	1	0.1%	52	1.9%	60	2.2%	55	2.0%
Education								
Elementary or Junior high school	1	0.1%	49	1.8%	50	1.8%	56	2.0%
High school	219	20.9%	782	28.7%	773	28.2%	715	26.1%
College	276	26.3%	571	21.0%	578	21.1%	559	20.4%
University	529	50.4%	1176	43.2%	1192	43.5%	1284	46.8%
Postgraduate	25	2.4%	145	5.3%	142	5.2%	124	4.5%
Other	0	0.0%	2	0.1%	4	0.2%	4	0.2%
Marital status								
Unmarried	399	38.0%	1228	45.1%	139	5.1%	1241	45.3%
Married	592	56.4%	1315	48.3%	412	15.0%	1344	49.0%
Divorced/Bereaved	59	5.6%	182	6.7%	570	20.8%	157	5.7%
Household income (JPY 1mil)								
< 1	7	0.7%	145	5.3%	437	16.0%	126	4.6%
1 < = < 3	77	7.3%	390	14.3%	369	13.5%	390	14.2%
3 < = < 5	239	22.8%	666	24.4%	208	7.6%	642	23.4%
5 < = < 7	228	21.7%	400	14.7%	50	1.8%	410	15.0%
7 < = < 10	256	24.4%	368	13.5%	31	1.1%	407	14.8%
10 < = < 15	137	13.1%	202	7.4%	523	19.1%	212	7.7%
15 < = < 20	33	3.1%	52	1.9%	52	1.9%	45	1.6%
20 > =	15	1.4%	34	1.3%	34	1.3%	28	1.0%
Unknown	58	5.5%	468	17.2%	468	17.2%	482	17.6%

### cTTO

**Fig. 1:**
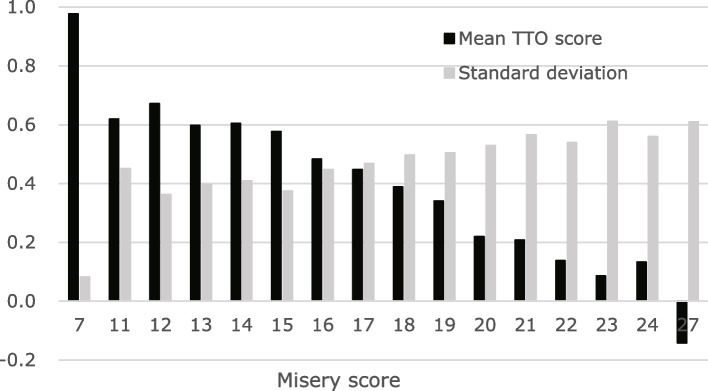
The relation between utility and severity of health states

**Fig. 2:**
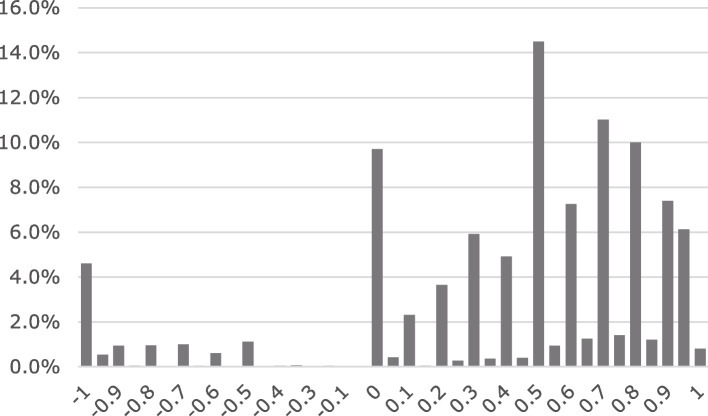
Distribution of cTTO responses

The 1,050 respondents collectively yielded 8,400 TTO data points. The TTO score for the health state [2222222] was 0.79 (highest) excluding health state [1111111], and the score for the health state [4444444] was -0.14 (lowest) (Additional file 1). In the task of evaluating health state [4444444], 47 respondents (62.7%) preferred the worst state (4,444,444) to death and 28 (37.3%) evaluated it as worse than death (WTD). Considering all responses, only 10.1% (*N* = 849) were evaluated as WTD health states. As the misery score (the sum of level scores across dimensions) increased, the mean cTTO value decreased, and the standard deviation increased with the misery score (Fig. 1). Figure 2 shows the distribution of the cTTO values. The peak of the distribution was at cTTO score = 0.5, and in regard to the distribution, the density of cTTO score < 0 was very low.

Table 2(a) presents the coefficients of the analysis obtained using the inconsistent and consistent models. One inconsistency (the second level of energy (EN)) was observed in the simple linear mixed models (model 1, model 3 and model 4), and the level was combined with the first level (model 2). However, the results of the Tobit model did not reveal any inconsistency. No significant interactions were observed in model 3 and model 4. The estimated utility values for the worst health state [4444444] were -0.02 (model 1) and -0.101 (model 4).

**Table 2:** Estimated coefficients by TTO and DCE

(a) cTTO																			
		Model 1			Model 2			Model 3			Model 4			Model 5					
		Unconstrained			Constrained			N4			N34			Tobit					
		Coef	SE	*P* value	Coef	SE	*P* value	Coef	SE	*P* value	Coef	SE	*P* value	Coef		SE		*P* value	
Intercept		0.067	0.02	0.006	0.066	0.02	0.005	0.071	0.025	0.005	0.074	0.025	0.003	0.010		0.025		0.701	
PD	2	0.025	0.02	0.088	0.025	0.02	0.088	0.025	0.015	0.088	0.025	0.015	0.088	0.036		0.015		0.016	
	3	0.178	0.02	< .0001	0.178	0.02	< .0001	0.178	0.015	< .0001	0.176	0.015	< .0001	0.189		0.015		< .0001	
	4	0.183	0.02	< .0001	0.183	0.02	< .0001	0.181	0.015	< .0001	0.181	0.015	< .0001	0.193		0.015		< .0001	
MH	2	0.022	0.02	0.128	0.022	0.02	0.128	0.023	0.015	0.122	0.022	0.015	0.128	0.033		0.015		0.028	
	3	0.098	0.02	< .0001	0.098	0.02	< .0001	0.099	0.015	< .0001	0.097	0.015	< .0001	0.109		0.015		< .0001	
	4	0.117	0.02	< .0001	0.117	0.02	< .0001	0.115	0.015	< .0001	0.115	0.015	< .0001	0.127		0.015		< .0001	
EN	2	-0.003	0.02	0.857	0	-	-	-0.003	0.015	0.857	-0.003	0.015	0.857	0.008		0.015		0.586	
	3	0.068	0.02	< .0001	0.07	0.01	< .0001	0.069	0.015	< .0001	0.067	0.015	< .0001	0.079		0.015		< .0001	
	4	0.09	0.02	< .0001	0.092	0.01	< .0001	0.089	0.015	< .0001	0.089	0.015	< .0001	0.101		0.015		< .0001	
MO	2	0.071	0.02	< .0001	0.071	0.02	< .0001	0.071	0.015	< .0001	0.071	0.015	< .0001	0.081		0.015		< .0001	
	3	0.176	0.02	< .0001	0.176	0.02	< .0001	0.177	0.015	< .0001	0.174	0.015	< .0001	0.186		0.015		< .0001	
	4	0.18	0.02	< .0001	0.18	0.02	< .0001	0.179	0.015	< .0001	0.178	0.015	< .0001	0.190		0.015		< .0001	
WS	2	0.023	0.02	0.117	0.023	0.02	0.117	0.024	0.015	0.107	0.023	0.015	0.117	0.033		0.015		0.025	
	3	0.11	0.02	< .0001	0.11	0.02	< .0001	0.110	0.015	< .0001	0.109	0.015	< .0001	0.121		0.015		< .0001	
	4	0.126	0.02	< .0001	0.126	0.02	< .0001	0.125	0.015	< .0001	0.124	0.015	< .0001	0.137		0.015		< .0001	
II	2	0.033	0.02	0.024	0.033	0.02	0.024	0.033	0.015	0.023	0.033	0.015	0.024	0.043		0.015		0.003	
	3	0.117	0.02	< .0001	0.117	0.02	< .0001	0.116	0.015	< .0001	0.115	0.015	< .0001	0.127		0.015		< .0001	
	4	0.137	0.02	< .0001	0.137	0.02	< .0001	0.135	0.015	< .0001	0.135	0.015	< .0001	0.147		0.015		< .0001	
BO	2	0.013	0.02	0.38	0.013	0.02	0.38	0.013	0.015	0.380	0.013	0.015	0.380	0.023		0.015		0.114	
	3	0.157	0.02	< .0001	0.157	0.02	< .0001	0.156	0.015	< .0001	0.155	0.015	< .0001	0.167		0.015		< .0001	
	4	0.185	0.02	< .0001	0.185	0.02	< .0001	0.184	0.015	< .0001	0.184	0.015	< .0001	0.196		0.015		< .0001	
N4								0.011	0.020	0.596									
N34											0.050	0.042	0.233						
Number of observations		8400																	
Log likelihood		-5736.3			-5733.0			-5739.2			-5737.900			-5706.3					
Number of inconsistencies		1			1			1			1			0					
(b) Simple DCE																			
		Model 6					Model 7					Model 8				Model 9			
		Conditional logit					N4					N34				Panel logit			
		Coef	SE		*P* value		Coef	SE		*P* value		Coef		SE	*P* value	Coef		SE	*P* value
PD	2	-0.294	0.023		< .0001		-0.293	0.023		< .0001		-0.292		0.023	< .0001	-0.176		0.026	< .0001
	3	-0.857	0.022		< .0001		-0.857	0.022		< .0001		-0.858		0.022	< .0001	-0.671		0.030	< .0001
	4	-1.107	0.023		< .0001		-1.111	0.023		< .0001		-1.107		0.023	< .0001	-0.813		0.039	< .0001
MH	2	-0.188	0.023		< .0001		-0.192	0.024		< .0001		-0.188		0.023	< .0001	-0.128		0.024	< .0001
	3	-0.570	0.024		< .0001		-0.573	0.024		< .0001		-0.570		0.024	< .0001	-0.512		0.025	< .0001
	4	-0.668	0.023		< .0001		-0.674	0.024		< .0001		-0.668		0.023	< .0001	-0.565		0.026	< .0001
EN	2	-0.040	0.026		0.118		-0.043	0.026		0.099		-0.042		0.026	0.110	-0.040		0.026	0.121
	3	-0.342	0.024		< .0001		-0.341	0.024		< .0001		-0.344		0.024	< .0001	-0.338		0.024	< .0001
	4	-0.497	0.025		< .0001		-0.501	0.025		< .0001		-0.499		0.025	< .0001	-0.491		0.025	< .0001
MO	2	-0.388	0.025		< .0001		-0.389	0.025		< .0001		-0.387		0.026	< .0001	-0.335		0.026	< .0001
	3	-0.800	0.024		< .0001		-0.801	0.024		< .0001		-0.801		0.024	< .0001	-0.766		0.024	< .0001
	4	-0.965	0.028		< .0001		-0.970	0.028		< .0001		-0.965		0.028	< .0001	-0.939		0.028	< .0001
WS	2	-0.161	0.023		< .0001		-0.162	0.023		< .0001		-0.162		0.023	< .0001	-0.147		0.023	< .0001
	3	-0.457	0.024		< .0001		-0.457	0.024		< .0001		-0.457		0.024	< .0001	-0.464		0.024	< .0001
	4	-0.590	0.023		< .0001		-0.594	0.024		< .0001		-0.591		0.023	< .0001	-0.597		0.023	< .0001
II	2	-0.141	0.023		< .0001		-0.139	0.023		< .0001		-0.141		0.023	< .0001	-0.156		0.023	< .0001
	3	-0.341	0.024		< .0001		-0.339	0.024		< .0001		-0.341		0.024	< .0001	-0.365		0.025	< .0001
	4	-0.403	0.024		< .0001		-0.405	0.024		< .0001		-0.403		0.024	< .0001	-0.439		0.024	< .0001
BO	2	-0.261	0.024		< .0001		-0.261	0.024		< .0001		-0.260		0.024	< .0001	-0.262		0.024	< .0001
	3	-0.718	0.026		< .0001		-0.720	0.026		< .0001		-0.718		0.026	< .0001	-0.704		0.026	< .0001
	4	-0.948	0.025		< .0001		-0.953	0.026		< .0001		-0.948		0.025	< .0001	-0.966		0.026	< .0001
N4							0.025	0.031		0.412									
N34												0.042		0.107	0.693				
Number of observations		81,750																	
Log likelihood		-24,225.1					-24,224.7					-24,225.0				-24,184.0			
Number of inconsistencies		0					0					0				0			
(c) DCE with duration																			
		Model 10			Model 11			Model 12			Model 13			Model 14			Model 15		
		Conditional logit (Unconstraint)			N4			N34			Conditional logit (Constraint)			Panel conditional logit (Unconstraint)			Panel conditional logit (Constraint)		
		Coef	SE	*P* value	Coef	SE	*P* value	Coef	SE	*P* value	Coef	SE	*P* value	Coef	SE	*P* value	Coef	SE	*P* value
Time		0.372	0.009	< .0001	0.348	0.010	< .0001	0.371	0.009	< .0001	0.376	0.009	< .0001	0.351	0.009	< .0001	0.354	0.009	< .0001
PD x Time	2	-0.044	0.004	< .0001	-0.051	0.004	< .0001	-0.043	0.004	< .0001	-0.043	0.004	< .0001	-0.021	0.005	< .0001	-0.020	0.004	< .0001
	3	-0.083	0.005	< .0001	-0.079	0.005	< .0001	-0.081	0.005	< .0001	-0.085	0.005	< .0001	-0.052	0.005	< .0001	-0.051	0.005	< .0001
	4	-0.109	0.004	< .0001	-0.106	0.005	< .0001	-0.106	0.005	< .0001	-0.110	0.004	< .0001	-0.061	0.005	< .0001	-0.060	0.005	< .0001
AD x Time	2	-0.058	0.005	< .0001	-0.059	0.005	< .0001	-0.060	0.005	< .0001	-0.059	0.005	< .0001	-0.052	0.005	< .0001	-0.053	0.005	< .0001
	3	-0.102	0.004	< .0001	-0.099	0.004	< .0001	-0.102	0.004	< .0001	-0.102	0.004	< .0001	-0.077	0.005	< .0001	-0.077	0.005	< .0001
	4	-0.111	0.006	< .0001	-0.100	0.006	< .0001	-0.112	0.006	< .0001	-0.113	0.005	< .0001	-0.085	0.006	< .0001	-0.085	0.006	< .0001
EN x Time	2	0.008	0.005	0.121	0.006	0.005	0.253	0.009	0.005	0.070	0.000			0.005	0.005	0.319	0.000		
	3	-0.032	0.004	< .0001	-0.031	0.004	< .0001	-0.031	0.004	< .0001	-0.036	0.004	< .0001	-0.041	0.004	< .0001	-0.043	0.004	< .0001
	4	-0.037	0.005	< .0001	-0.028	0.005	< .0001	-0.036	0.005	< .0001	-0.040	0.004	< .0001	-0.041	0.005	< .0001	-0.044	0.004	< .0001
MO x Time	2	-0.043	0.004	< .0001	-0.042	0.004	< .0001	-0.042	0.004	< .0001	-0.043	0.004	< .0001	-0.048	0.004	< .0001	-0.048	0.004	< .0001
	3	-0.072	0.004	< .0001	-0.076	0.004	< .0001	-0.071	0.004	< .0001	-0.074	0.004	< .0001	-0.081	0.004	< .0001	-0.082	0.004	< .0001
	4	-0.117	0.004	< .0001	-0.114	0.004	< .0001	-0.115	0.004	< .0001	-0.119	0.004	< .0001	-0.125	0.004	< .0001	-0.126	0.004	< .0001
WS x Time	2	-0.010	0.005	0.036	-0.001	0.005	0.877	-0.011	0.005	0.020	-0.010	0.005	0.029	-0.019	0.005	< .0001	-0.019	0.005	< .0001
	3	-0.057	0.005	< .0001	-0.053	0.005	< .0001	-0.058	0.005	< .0001	-0.055	0.005	< .0001	-0.070	0.005	< .0001	-0.069	0.005	< .0001
	4	-0.080	0.005	< .0001	-0.067	0.005	< .0001	-0.081	0.005	< .0001	-0.078	0.005	< .0001	-0.088	0.005	< .0001	-0.087	0.005	< .0001
SR x Time	2	-0.013	0.005	0.006	-0.007	0.005	0.125	-0.013	0.005	0.003	-0.013	0.005	0.004	-0.021	0.005	< .0001	-0.021	0.005	< .0001
	3	-0.072	0.005	< .0001	-0.065	0.005	< .0001	-0.072	0.005	< .0001	-0.069	0.004	< .0001	-0.071	0.005	< .0001	-0.071	0.005	< .0001
	4	-0.064	0.005	< .0001	-0.049	0.005	< .0001	-0.063	0.005	< .0001	-0.069	0.004	< .0001	-0.074	0.005	< .0001	-0.074	0.005	< .0001
BO x Time	2	-0.053	0.005	< .0001	-0.054	0.005	< .0001	-0.055	0.005	< .0001	-0.049	0.005	< .0001	-0.047	0.005	< .0001	-0.046	0.005	< .0001
	3	-0.108	0.004	< .0001	-0.100	0.005	< .0001	-0.106	0.004	< .0001	-0.107	0.004	< .0001	-0.110	0.004	< .0001	-0.110	0.004	< .0001
	4	-0.132	0.004	< .0001	-0.128	0.004	< .0001	-0.131	0.004	< .0001	-0.129	0.004	< .0001	-0.127	0.004	< .0001	-0.127	0.004	< .0001
N4 x Time					-0.048	0.006	< .0001												
N34 x Time								-0.057	0.019	0.003									
Number of observations		82,170												82,170					
Log likelihood		-28,478.0			-28,478.0			-28,478.0			-25,969.8			-25,803.6			-25,804.1		
Number of inconsistencies		2			2			2			0			1			1		
(d) Ternary DCE																			
		Model 16			Model 17			Model 18			Model 19			Model 20			Model 21		
		Conditional logit (Unconstraint)			N4			N34			Conditional logit (Constraint)			Panel conditional logit (Unconstraint)			Panel conditional logit (Constraint)		
		Coef	SE	*P* value	Coef	SE	*P* value	Coef	SE	*P* value	Coef	SE	*P* value	Coef	SE	*P* value	Coef	SE	*P* value
Time		0.298	0.006	< 0.001	0.275	0.006	< .0001	0.291	0.006	< .0001	0.304	0.005	< 0.001	0.197	0.006	0.000	0.197	0.006	0.000
PD x Time	2	-0.028	0.003	< 0.001	-0.031	0.003	< .0001	-0.025	0.003	< .0001	-0.026	0.003	< 0.001	0.004	0.003	0.286	0.000		
	3	-0.054	0.003	< 0.001	-0.052	0.003	< .0001	-0.051	0.003	< .0001	-0.053	0.003	< 0.001	-0.015	0.003	0.000	-0.015	0.002	0.000
	4	-0.082	0.003	< 0.001	-0.078	0.003	< .0001	-0.078	0.003	< .0001	-0.081	0.003	< 0.001	-0.012	0.003	0.000	-0.015		
AD x Time	2	-0.038	0.004	< 0.001	-0.038	0.004	< .0001	-0.040	0.004	< .0001	-0.039	0.004	< 0.001	-0.012	0.004	0.000	-0.012	0.003	0.000
	3	-0.054	0.003	< 0.001	-0.050	0.003	< .0001	-0.054	0.003	< .0001	-0.055	0.003	< 0.001	-0.013	0.003	0.000	-0.013	0.003	0.000
	4	-0.079	0.004	< 0.001	-0.071	0.004	< .0001	-0.080	0.004	< .0001	-0.080	0.004	< 0.001	-0.030	0.004	0.000	-0.029	0.004	0.000
EN x Time	2	0.005	0.003	0.168	0.001	0.003	0.772	0.008	0.003	0.020	0.000			0.000	0.003	0.938	0.000		
	3	0.001	0.003	0.755	0.000	0.003	0.901	0.003	0.003	0.279	-0.002	0.003	0.408	-0.024	0.003	0.000	-0.023	0.002	0.000
	4	-0.015	0.003	< 0.001	-0.009	0.003	0.010	-0.012	0.003	0.000	-0.017	0.003	< 0.001	-0.024	0.003	0.000	-0.023		
MO x Time	2	-0.037	0.003	< 0.001	-0.035	0.003	< .0001	-0.034	0.003	< .0001	-0.036	0.003	< 0.001	-0.029	0.003	0.000	-0.029	0.003	0.000
	3	-0.072	0.003	< 0.001	-0.075	0.003	< .0001	-0.070	0.003	< .0001	-0.073	0.003	< 0.001	-0.073	0.003	0.000	-0.073	0.003	0.000
	4	-0.078	0.003	< 0.001	-0.073	0.003	< .0001	-0.074	0.003	< .0001	-0.078	0.003	< 0.001	-0.084	0.003	0.000	-0.084	0.003	0.000
WS x Time	2	0.007	0.003	0.038	0.014	0.003	< .0001	0.006	0.003	0.083	0.000			-0.016	0.003	0.000	-0.014	0.003	0.000
	3	-0.011	0.003	0.001	-0.007	0.003	0.035	-0.012	0.003	0.000	-0.015	0.003	< 0.001	-0.041	0.003	0.000	-0.039	0.003	0.000
	4	-0.018	0.003	< 0.001	-0.008	0.003	0.014	-0.019	0.003	< .0001	-0.021	0.003	< 0.001	-0.039	0.003	0.000	-0.039		
SR x Time	2	-0.025	0.003	< 0.001	-0.023	0.003	< .0001	-0.027	0.003	< .0001	-0.024	0.003	< 0.001	-0.022	0.003	0.000	-0.022	0.003	0.000
	3	-0.042	0.003	< 0.001	-0.036	0.003	< .0001	-0.041	0.003	< .0001	-0.042	0.003	< 0.001	-0.027	0.003	0.000	-0.026	0.003	0.000
	4	-0.043	0.003	< 0.001	-0.033	0.004	< .0001	-0.041	0.003	< .0001	-0.044	0.003	< 0.001	-0.047	0.003	0.000	-0.047	0.003	0.000
BO x Time	2	-0.015	0.003	< 0.001	-0.013	0.003	0.000	-0.018	0.003	< .0001	-0.015	0.003	< 0.001	-0.007	0.003	0.048	-0.007	0.003	0.044
	3	-0.051	0.003	< 0.001	-0.046	0.003	< .0001	-0.048	0.003	< .0001	-0.052	0.003	< 0.001	-0.061	0.003	0.000	-0.061	0.003	0.000
	4	-0.076	0.003	< 0.001	-0.070	0.003	< .0001	-0.075	0.003	< .0001	-0.076	0.003	< 0.001	-0.076	0.003	0.000	-0.075	0.003	0.000
N4 x Time					-0.042	0.004	< .0001												
N34 x Time								-0.116	0.014	< .0001									
Number of observations		205,650												205,650					
Log likelihood		-73,695.1			-73,695.1			-73,695.1			-69,744.3			-67,443.7			-67,445.0		
Number of inconsistencies		2			2			2			0			5			0		

*PD* pain/discomfort, *MH* mental health, *EN* energy, *MO* mobility, *WS* work/school, *II* interpersonal interactions, *BO* burden to others

### Simple DCE

**Fig. 3:**
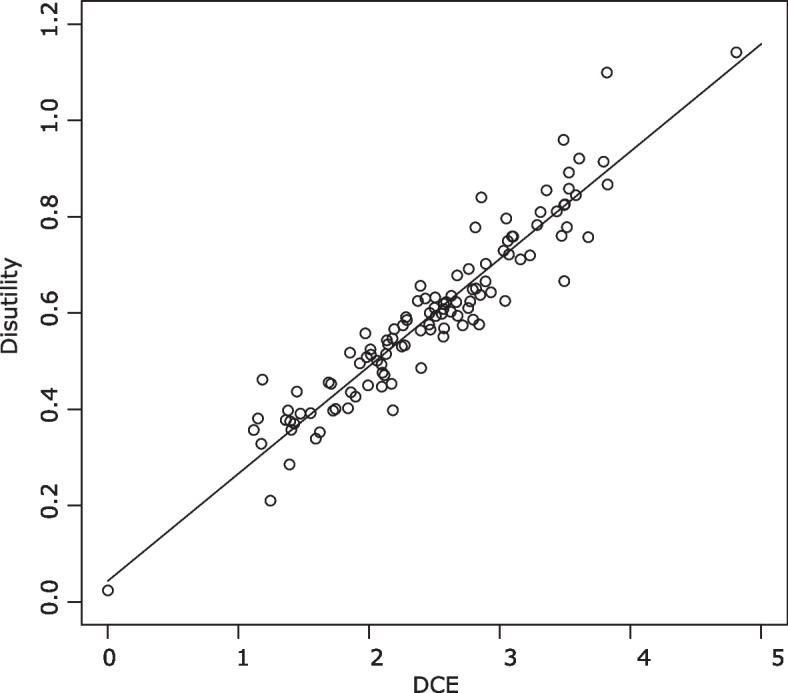
The relation between cTTO and DCE disutility

Table 2(b) presents the parameter estimates obtained from the DCE data. No inconsistencies were observed between groups in any of the models. No significant interactions were observed in model 3 and model 4. Using the coefficients of model 8 in Table 2(b), latent DCE scores were computed for the AP-7D states, because the AIC of model 8 was the smallest. The linear relation was estimated to predict the cTTO values based on the latent DCE values. The estimated equation from the regression of the cTTO score (disutility) to the latent DCE score was 1- cTTO score (disutility) = 0.223*x + 0.0433, where x denotes the latent DCE score. The DCE coefficients were rescaled using this equation. Figure 3 shows the relationship between the observed disutility and the derived DCE values. The fitting of the linear regression seems satisfactory.

### DCE with duration and ternary DCE

Table 2(c) shows the results for the DCE with duration and ternary DCE methods. The estimated coefficients using a simple conditional logit model showed two inconsistencies (model 10 to model 12), and levels 1 and 2 of EN and levels 3 and 4 of II were combined. The results by the panel conditional logit model (model 14) showed only one inconsistency. Similarly, two inconsistencies were observed in the coefficients of ternary DCE in model 16 to model 18 shown in Table 2(d). Therefore, levels 1 and 2 of EN and levels 1 and 2 of WS were combined. In contrast to DCE with duration data, the panel conditional logit model (model 20) showed an increased number of inconsistencies, although the AIC of model 20 was smaller than that of model 16.

### Comparison of the value sets derived from the four cTTO and DCE approaches

**Fig. 4:**
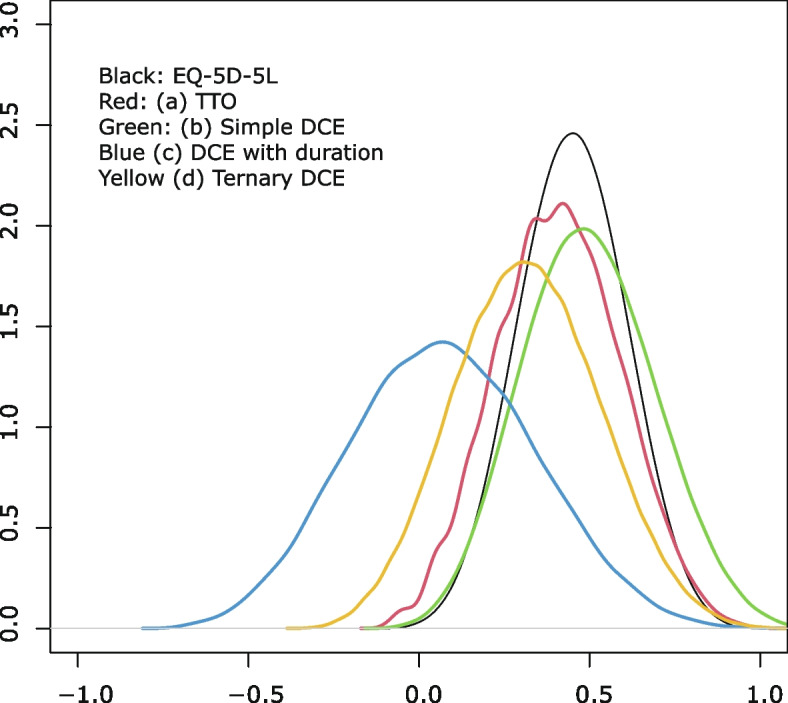
Distribution of the Japanese EQ-5D-5L and AP-7D

**Fig. 5:**
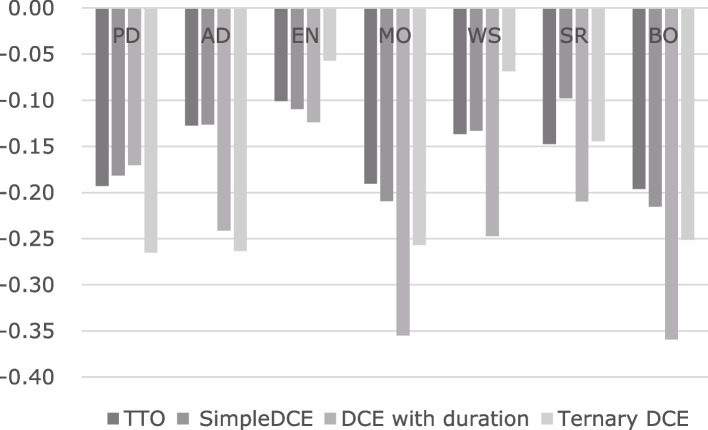
Coefficients of the worst level (level 4) obtained using the four valuation methods

Table 3 lists the anchored results obtained using the coefficients from the constrained models (model 5, model 9, model 15, and model 19). The selections from some models were determined mainly considering the number of inconsistencies and AIC. Simpler models without interactions were preferred if the characteristics of the value sets were not significantly different. Table 3 can be used to calculate the utility from the health states using AP-7D. The minimum values of the models were as follows: TTO model, -0.101; simple DCE model, -0.116; DCE with duration model, -0.706; and ternary DCE model, -0.306. The score estimated using the DCE with the duration model was much lower than the scores estimated using the other models. Figure 4 shows the distribution of the utility of all the health states described by AP-7D and the Japan EQ-5D-5L. As compared with the EQ-5D-5L based on the Japanese value set, all the scores on the Japanese AP-7D had moved to the left. The distributions of the results obtained using the TTO and simple DCE tasks overlapped. Those obtained using the ternary DCE method were distributed between the results obtained with the simple DCE and DCE with duration methods. Figure 5 compares the coefficients of the worst level (level 4) by the four valuation methods. BO showed one of the largest decrements in the seven domains.

**Table 3:** Scoring algorithm for AP-7D by all four models

		TTO	Simple DCE	DCE with duration	Ternary DCE
Intercept		-0.010	-0.043		
PD	2	-0.036	-0.039	-0.057	-0.086
	3	-0.189	-0.150	-0.146	-0.175
	4	-0.193	-0.181	-0.170	-0.265
MH	2	-0.033	-0.029	-0.149	-0.128
	3	-0.109	-0.114	-0.218	-0.181
	4	-0.127	-0.126	-0.241	-0.263
EN	2	-0.008	-0.009	0.000	0.000
	3	-0.079	-0.075	-0.122	-0.007
	4	-0.101	-0.110	-0.124	-0.057
MO	2	-0.081	-0.075	-0.136	-0.119
	3	-0.186	-0.171	-0.231	-0.240
	4	-0.190	-0.209	-0.355	-0.257
WS	2	-0.033	-0.033	-0.053	0.000
	3	-0.121	-0.104	-0.195	-0.050
	4	-0.137	-0.133	-0.247	-0.069
II	2	-0.043	-0.035	-0.059	-0.080
	3	-0.127	-0.081	-0.201	-0.138
	4	-0.147	-0.098	-0.210	-0.144
BO	2	-0.023	-0.059	-0.130	-0.051
	3	-0.167	-0.157	-0.311	-0.171
	4	-0.196	-0.215	-0.359	-0.251
minimum value		-0.101	-0.116	-0.706	-0.306

*PD* pain/discomfort, *MH* mental health, *EN* energy, *MO* mobility, *WS* work/school, *II* interpersonal interactions, *BO* burden to others

## Discussion

We constructed four value sets for AP-7D. All models, except simple DCE, showed some inconsistencies (the second level of EN in cTTO, DCE with duration and ternary DCE, and the second level of WS in the ternary DCE), but the number was limited. Therefore, a constrained model was constructed and the first preliminarily value sets for AP-7D in Japan were calculated.

As shown in Fig. 5, EN was among the domains with the least influence on the AP-7D score in all four models. Especially, the second level of EN did not have any significant negative preferences as compared with the first, except in the DCE with mapping model. In contrast, PD, MO, and BO had the largest coefficients on the scoring algorithm in all models. It is noteworthy that BO showed the largest impact on preferences, similar to PD and MO. We think that this result may reflect the characteristics of Japanese people who hesitate much before troubling another. The influences of MH, WS, and II differed depending on the model. The coefficient of MH was larger in the DCE with duration and ternary DCE models than in the TTO and DCE with mapping models. The influence of the WS domain was similar to that of the EN domain in the ternary DCE model. The scoring algorithm drawn using the DCE with mapping model showed that the coefficients of domain II were the smallest, which implies that it was smaller than that of the EN domain. The importance of some domains differed among the models.

We used four valuation methods to construct a value set for AP-7D. The minimum value was the highest (-0.101) by the TTO model and lowest by the DCE with duration (-0.706). EQ-5D-5L was also valued by the cTTO method, and the utility of the worst health state by the Japanese EQ-5D-5L was -0.025 [22], which is the highest recorded value in the world. Japanese people have a strong risk-averse feeling about death and are reluctant to trade health states with death. Therefore, the Japanese TTO-based value set may overestimate the utility of each health state. In contrast, the utility scores obtained using DCE with duration were very low. This means that Japanese people willingly trade life-years with their health state, although they do not prefer death. It is difficult to interpret this; they may imagine that the reduction in life years is different from death. This means that the utility scores obtained using DCE with duration may underestimate the utility of the AP-7D health states. However, ternary DCE included the “immediate death” card. In the ternary tasks, the respondents traded health states with death, although they responded to DCE with duration tasks. The value set obtained using the ternary DCE method showed intermediate characteristics between the value sets obtained using the cTTO and DCE with duration tasks.

However, the Japanese guidelines for economic evaluation recommend using EQ-5D-5L (“8.2.1 If Japanese QoL scores (utilities) are newly collected for a cost-effectiveness analysis, EQ-5D-5L is recommended as the first choice.”). For example, the NICE in the UK and HAS in France also require the submission of EQ-5D-based utility scores. It may be important that new instruments are valuated using a similar cTTO-based method as the EQ-5D-5L.

One limitation of this study was the sampling method. Neither face-to-face nor web surveys allow respondents to be chosen randomly across Japan. Although the major background factors of the respondents are similar to those of the Japanese population, the influence of the sampling method may not be negligible. In addition, our sample was limited to people aged 20–69 years because of the outbreak of COVID-19. We recognize it is better to include more elderly people in our survey. The inclusion criteria of respondents has to be reconsidered when actual valuation survey is performed. Face-to-face survey was used only for the TTO survey. Difference in the survey mode could have influenced the results. Additionally, the survey was limited to Japan. It is unclear whether our findings and discussions can be generalized to other countries. Moreover, the influence of the COVID-19 outbreak, which could have changed the preferences for health states, is unknown. Elderly people could not be recruited into this survey because they were a high-risk population for COVID-19.

## Conclusion

We constructed and compared four value sets for the Japanese AP-7D, which paves the way for considering valuation methods for an international AP-7D valuation survey. To reflect people’s preferences more appropriately for effective decision making, we have to consider the methods to be applied. As discussed above, the value sets are completely different depending on the valuation methods, especially in the range of the measurement (negative utility scores). In addition, our findings could contribute to the development of not only AP-7D, but also other PBMs. The choice of “immediate death” significantly impacts the results, and the degree of death-risk acceptance may differ among countries, reflecting their respective cultures. Our goal is to show this instrument as a good alternative to existing PBMs, such as EQ-5D, SF-6D, and HUI. This is the first step of our future plan to improve decision making. To select the most appropriate valuation methods, we require more qualitative and deliberate processes with expert as well as non-expert members. The input of actual decision makers may also be required.

## Supplementary Information

**Additional file 1.**

## Data Availability

The datasets generated and/or analyzed during the current study are not publicly available as consent for the same was not obtained from the participants, but will be made available by the corresponding author upon reasonable request.

## Abbreviations

AP-7D: Asia-preference-based measure-7 dimension
BO: Burden to others
CAPI: Computer-assisted personal interviewing
DCE: Discrete choice experiment
EN: Energy
HTA: Health technology assessment
II: Interpersonal interactions
MH: Mental health
MO: Mobility
PBM: Preference-based measures
PD: Pain/discomfort
QALY: Quality-adjusted life-year
TTO: Time-trade-off
WS: Work/school
