# Supplementary material for: Comparison of four value sets derived using different TTO and DCE approaches: application to the new region-specific PBM, AP-7D
Source: Health Qual Life Outcomes. 2024 Feb 10;22:16. doi: 10.1186/s12955-024-02233-2 (PMC11380401; doi:10.1186/s12955-024-02233-2)
Supplement: Supplementary file 1 — Additional file 1. [file 12955_2024_2233_MOESM1_ESM.docx]

**Asian preference-based measure 7 dimensions (AP-7D) instrument**

Think about your health during the past week. Please select the box that best applies to you. Health includes both physical and mental health.

|  | Not at all | A  little | Quite  a bit | Very much |
| --- | --- | --- | --- | --- |
| 1. I was in pain or discomfort and it prevented me from doing what I wanted to do. | □ | □ | □ | □ |
| 2. I was anxious or depressed. | □ | □ | □ | □ |
| 3. I lacked the energy to do things. | □ | □ | □ | □ |
| 4. I had difficulty walking (or moving with the support of a wheelchair). | □ | □ | □ | □ |
| 5. My health affected my ability to work (outside or inside the home) or go to school | □ | □ | □ | □ |
| 6. Because of my health, I had less interaction with family, close friends, and such. | □ | □ | □ | □ |
| 7. Because of my health, I felt I was a burden to others. | □ | □ | □ | □ |


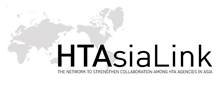

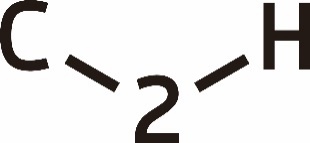


This instrument was co-developed by Center for Outcomes Research and Economic Evaluation for Health (C2H) and HTAsiaLink. ©C2H
